# Glacier retreat triggers changes in biodiversity and plant–pollinator interaction diversity

**DOI:** 10.1007/s00035-024-00309-9

**Published:** 2024-04-09

**Authors:** Bao Ngan Tu, Nora Khelidj, Pierfilippo Cerretti, Natasha de Vere, Andrea Ferrari, Francesco Paone, Carlo Polidori, Jürg Schmid, Daniele Sommaggio, Gianalberto Losapio

**Affiliations:** 1 Institute of Earth Surface Dynamics, University of Lausanne, 1015 Lausanne, Switzerland; 2 Department of Biosciences, University of Milan, 20133 Milan, Italy; 3 Department of Biology and Biotechnologies, Sapienza University of Rome, 00185 Rome, Italy; 4 Natural History Museum of Denmark, University of Copenhagen, Øster Voldgade 5-7, 1350 Copenhagen K, Denmark; 5 Department of Environmental Science and Policy, University of Milan, 20133 Milan, Italy; 6 Ilanz, Switzerland; 7 Department of Life Sciences, University of Modena and Reggio Emilia, 42121 Reggio Emilia, Italy

**Keywords:** Alpine plants, Biodiversity change, Ecological networks, Glacier exctinction, Global warming, Mont Miné glacier foreland, Mutualistic networks, Species interactions, Pollinators

## Abstract

Due to global warming, the worldwide retreat of glaciers is causing changes in species diversity, community composition, and species interactions. However, the impact of glacier retreat on interaction diversity and ecological networks remains poorly understood. An integrative understanding of network dynamics may inform conservation actions that support biodiversity and ecosystem functioning after glacier extinction. Here, we address how glacier retreat affects the frequency, diversity, and complexity of plant–pollinator interactions, both directly and indirectly through biodiversity change. We surveyed flower visitors (pollinators) and analyzed pollination networks across a gradient of 170 years of glacier retreat (Mont Miné glacier, Valais, Switzerland) which ranges from patchy grasslands to closed forests. We reported a strong impact of glacier retreat on both plant and pollinator communities. Notably, the diversity of plant–pollinator interactions was sharply affected by glacier retreat: interaction diversity increased few years after glacier retreat, but it ultimately decreased in late stages dominated by forests. In contrast, we found that plant–pollinator network complexity did not change with glacier retreat. Our results indicate that the development of plant–pollinator networks is a two-phases process. In the first phase, glacier retreat makes space to plant colonization. This initial increase in plant diversity drives the increase in pollinator and interaction diversity. The second phase is characterized by turnover as woody species encroaches and dominates the community, decreasing the diversity of plant species in ultimate instance. The local decrease of plant diversity leads to a local decrease in pollinator and interaction diversity. Slowing down woody encroachment and enhancing flower diversity, which is initially supported by the glacial landscape, may be key strategies for halting the erosion of ecological networks while increasing biodiversity and ecosystem functioning. Our research thus can help resolve the overarching question of how to conserve ecosystems once glaciers are extinct, pointing toward a composite role of both habitat structure and biological functions.

**Supplementary Information:**

The online version contains supplementary material available at 10.1007/s00035-024-00309-9.

## Introduction

Greenhouse gas emissions are altering climate patterns and warming up the Planet (IPCC 2022). As temperature increases globally at unprecedented rates in human history, glaciers are retreating and disappearing worldwide (Roe et al. 2017). Glaciers have been retreating during the last two decades twice as fast as ever observed in the last two centuries. The recent IPCC Sixth Assessment Report (IPCC 2022) highlights that glaciers are unique and threatened ecological and human systems that are a major reason for concern. Current projections indicate that, without drastic changes or measures, if the melting trend of the last 20 years continues, almost half (46%) of the ice volume in the Alps will disappear by 2050 (Cook et al. 2023). Yet, the consequences of glacier retreat for biological interactions remain poorly understood.

Glacier retreat affects landscape composition, soil properties, water resources, micro- and macro-climate (Fell et al. 2017; Brighenti et al. 2019). Notably, glacier retreat drives biodiversity change, i.e., changes in the diversity and composition of biological communities. As glaciers retreat, new terrains (hereafter glacier forelands; Matthews 1992) are exposed to colonization by living organisms, primarily plants, making space to species colonization and primary succession (Whittaker 1993; Burga et al. 2010; Tampucci et al. 2015; Cauvy-Fraunié and Dangles 2019; Fickert 2020; Ficetola et al. 2021). Following pioneering plants, insects take advantage of the newly created habitats and the increased availability and diversity of plant resources (Losapio et al. 2015, 2016; Tampucci et al. 2015; Junker et al. 2020). Previous studies indicate that species richness increases with glacier retreat albeit for a relatively brief period (Burga et al. 2010; Cauvy-Fraunié and Dangles 2019; Fickert 2020), Moreover, the new ice-free terrains could create refugia for high alpine plants shifting their distributions due to climate change (Gentili et al. 2020). But this trend holds true only as long as glaciers are still present in the landscape (Stibal et al. 2020; Losapio et al. 2021a; Anthelme et al. 2022).

As the succession proceeds, species turnover and competition can prevail over colonization and facilitation, which lead to local biodiversity decline (Anthelme et al. 2021; Losapio et al. 2021a; Erschbamer et al. 2023). With the extinction of glaciers from the landscape, the vegetation attains a stage typical for the specific elevation, resulting in diminished biodiversity. Consequently, in a scenario where glaciers remain intact without retreating, there would be neither a primary succession nor an upsurge in biodiversity. By contrast, due to global warming and current trends of glacier retreat, 11% of Arctic aquatic species (Fell et al. 2017) and 23% of Alpine plant species (Losapio et al. 2021a) may be threatened by local disappearance. Given the alarming rates of glacier retreat, specialist species inhabiting glacier ecosystems are at risk of extinction (Stibal et al. 2020; Losapio et al. 2021a). However, most of current research remains at a descriptive level—“counting the books while the library burns” (Lindenmayer et al. 2013)—that is cataloguing species and counting species numbers. Overlooking the causes of species decline and the mechanisms underlying biodiversity maintenance is hindering our ability to devise strategies for halting biodiversity loos and conserving biodiversity.

Biodiversity is more than a list of species (Bascompte and Jordano 2014). Plants play a key role in the process of ecosystem development by stabilizing and enriching soil (Matthews 1992). As primary producers, plants create the base of the food web which maintains other trophic levels such as pollinators and herbivores (Losapio et al. 2015; Inouye 2020). Plants have mutualistic interactions with pollinators as both partners gain benefits: pollinators disperse pollen for plants while plants provide food resources for pollinators (Calatayud et al. 2018). Without pollinators, plant populations, plant fitness, and productivity would face a large decrease (Losapio et al. 2016; Adedoja et al. 2018; Hanusch et al. 2022). Flies, bees, butterflies, moths, beetles, and ants are characteristic pollinators of high-altitude alpine plants (Inouye 2020; McCabe and Cobb 2021). Pollinator distribution and community assembly are mainly driven by environmental conditions during the initial stages of colonization (Whittaker 1993; Kaufmann 2001). Then, habitat creation and modification by plants as well as species interactions become more important for biodiversity maintenance and community dynamics (Kaufmann and Raffl 2002; Vater and Matthews 2013; Erschbamer and Caccianiga 2016; Losapio et al. 2016).

The process of ecosystem development after glacier retreat is accompanied by the formation of new and the loss of former plant–pollinator interactions (Albrecht et al. 2010; Losapio et al. 2015). This way, plant–pollinator networks are sensitive to changes in both environmental conditions and the distribution of interacting partners (Adedoja et al. 2018). Although plants and pollinators are among the primary pioneers in glacier forelands, research on plant and pollinator communities proceeded independently, focusing either on plants or on pollinators, but rarely on plant–pollinator interactions (Albrecht et al. 2010; Losapio et al. 2015). Little is known about the drivers of plant–pollinator interactions and the effects of glacier retreat on the structure of pollination networks.

Network theory and analysis are established tools for mapping the structure and dynamics of plant–pollinator interactions (Bascompte and Jordano 2014). Recent advances highlight an increase in the complexity of plant–pollinator interactions with glacier retreat (Albrecht et al. 2010; Losapio et al. 2015). This indicates that the dynamics of alpine plant and pollinator communities may be driven by mutualistic interactions more than just by abiotic factors (Losapio et al. 2016). Furthermore, the key role of plant is indicated by the evidence that plant diversity is a stronger predictor of network complexity and interaction diversity than pollinator diversity (Robinson et al. 2018). Yet, it is not clear how interaction diversity changes with glacier retreat. The lack of comprehensive network-level studies impairs our ability to predict the fate of biodiversity and the functioning of ecosystems on glacier foreland. An integrative understanding of the impact of glacier retreat on plant and pollinator communities and their interaction networks is therefore of major importance to biodiversity maintenance.

To address these challenges, we asked the following questions: (i) How does glacier retreat affect the diversity of plant and pollinator communities? (ii) How does plant diversity contribute to the change of pollinator diversity with glacier retreat? (iii) How does glacier retreat impact the complexity of plant–pollinator interactions? We hypothesize that the diversity of plant–pollinator interactions as well as network complexity increase only shortly after glacier retreat, while it will ultimately decrease in the long term.

## Materials and methods

### Study system

The study was performed along the foreland of Mont Miné glacier (Valais, Switzerland). Since the end of the Little Ice Age (*c* 1850), Mont Miné glacier has retreated by 2.53 km in length and 130 m in height as of 2019 (Nicolussi et al. 2022). Following glacier dynamics, we estimated the age of plant communities based on existing geochronology of Ferpeclé and Mont Miné glaciers (Lambiel et al. 2016; Nicolussi et al. 2022), and complemented it with our additional reconstruction based on historical cartography (https://map.geo.admin.ch) and field validation of moraine margins.

**Fig. 1:**
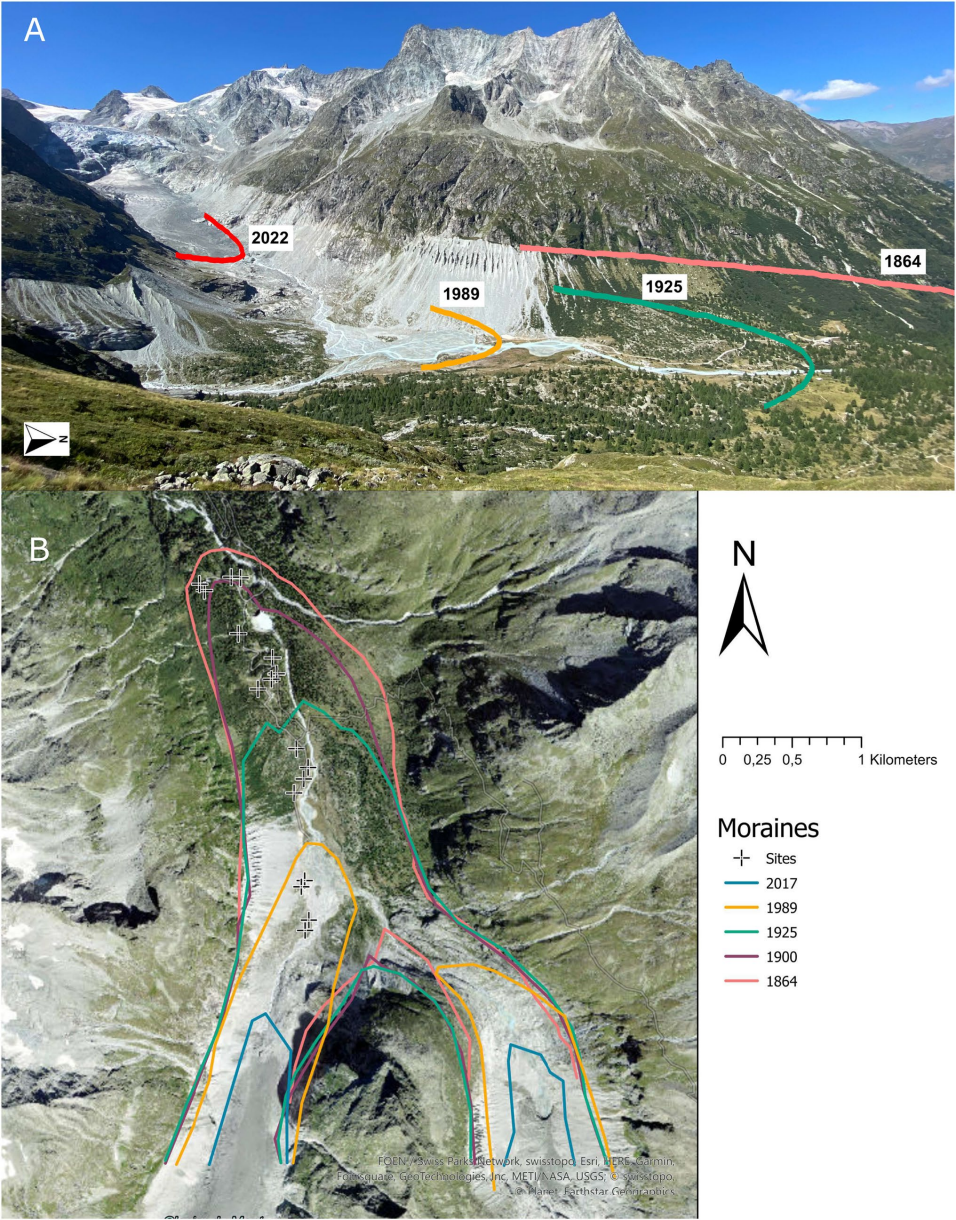
Study sites at Mont Miné glacier**,** Valais, Switzerland. **A** View of Mont Miné glacier with visible moraines (photo credit: Bao Ngan Tu). **B** Mont Miné and Ferpécle glacier foreland. Lines represent four main moraines deposited in 1864 (pink; S1), 1900 (purple; S2), 1925 (green; S3), 1989 (yellow; S4) and glacier extension in 2017 (blue) (photo credit: https://map.geo.admin.ch). Sampling locations are represented with + symbol

We divided the Mont Miné foreland into four glacier retreat stages delimited by moraines deposited in the following years: 1989 (S1), 1925 (S2), 1900 (S3) and 1864 (S4) (Fig. 1A). Such 170-years range of glacier retreat represents a gradient of ecosystem development ranging from patchy, pioneer grasslands to closed forests (Delarze et al. 2015; Price et al. 2021). Early stages include habitats typical of alluvial zones and moraines (habitat 3.2), debris and screes (habitat 3.3), and pioneer grasslands on rocky soil (habitat 4.1). Notably, pioneer grasslands are remarkably different from those grasslands outside the glacier foreland, whereas examined forests are structurally and compositionally similar to those forests outside the glacier foreland (Lambiel et al. 2016) (Fig. 1A). Plant species occurring in pioneer stages do not grow outside the glacier foreland (Delarze et al. 2015; Price et al. 2021). As the majority of those plant species are “glacier specialists”, they do not grow in the surrounding vegetation (Losapio et al. 2021a). Intermediate stages include mountain, nutrient-poor grasslands (habitat 4.3), snow beds (habitat 4.4), tall, nutrient-rich grasslands (habitat 5.2), bushes (habitat 5.3), and dwarf-shrub heat (habitat 5.4). Late stages include bushes (habitat 5.3) and mountain coniferous forests (habitat 6.6). We would like to stress the fact that stage four is the stable mountain coniferous forest of *Larix decidua*, *Picea abies*, *Rhododendron ferrugineum* and *Vaccinium myrtillus* that represents the “climax” vegetation and also occurs outside the glacier foreland (Delarze et al. 2015; Price et al. 2021).

We defined the age (*x*) of each stage (*i*) as the average years since glacier retreat. The age of each stage was then calculated between two adjacent moraines as:

$${x}_{i}= {x}_{0}-\frac{\left({x}_{old}+{x}_{young}\right)}{2}$$

where ${x}_{0}$ is the year of sampling (i.e., 2022), ${x}_{old}$ and ${x}_{young}$ is the year of older and younger moraines, respectively. Average ages of plant communities were then 17, 65, 110, 140 years for stage 1, stage 2, stage 3, and stage 4, respectively.

### Data collection

The sampling took place in during the summer 2022, throughout the flowering season, from mid-June until late July. Pollinators were sampled during sunny days, from 10am to 5 pm to cover their maximum activity. We set up four plots in each stage (*n* = 16) (Fig. 1B). Plots were randomly chosen on the right bank of Mont Miné glacier foreland. As this glacier foreland is flat, there are no differences in elevation among plots (range from 1961 to 2000 m a.s.l). Areas disturbed by hydropower activities were avoided.

In each permanent plot, we surveyed plant communities by recording species composition and estimating visually plant cover (accuracy 10%). Plant species were identified according to Flora Helvetica (Lauber et al. 2018; https://www.infoflora.ch). We recorded a total of 130 plant species belonging to 32 families (Table S1). Throughout the season, we also recorded phenology and flowering stage (i.e., buds, flowering, fruiting) of plant species at the time of sampling.

Plant–pollinator interactions were studied by sampling flower visitors (pollinators) on plants. We considered as pollinator any flying insect that gets in touch with flower reproductive parts. Sampling was carried out for a standard amount of observation time of 30 min per sampling round. In each plot, we adopted two complementary sampling methods: quadrats and transects (Gibson et al. 2011; Grange et al. 2021). Quadrats consisted of a of 3 × 3 m square surface with 1 m buffer, resulting in 25 m^2^ sampling area. Transects consisted of two orthogonal 25 m long and 1 m belt across the plot center, resulting in 50 m^2^ sampling area. The differences in survey area account for average differences in plant density as in these terrains transects contain less plants and have more bare ground (Gibson et al. 2011). Combining these two different survey methods helps us to maximize the variability of pollinator sampling.

Pollinators were sampled with an entomological aspirator and sweep net. To minimize the impact of our sampling on pollinator populations, we collected the minimum number of specimens, especially aculeata, to only those necessary for species identification. Then, we proceeded with sampling, recording, and releasing insects. For each pollinator sampled, we recorded the plant species of the flower it was found on. That is, pollination interactions were conducted at the species level. Sampling was randomly replicated throughout the season, making sure that each plot was sampled at different times of day. In total, we performed six rounds of sampling for each of the 16 plots, resulting in *n* = 96 replicates.

Pollinators were identified at the lowest taxonomic level, i.e., species, whenever possible. Bumblebees were identified following Rasmont et al. (2021) and Cappellari et al. (2018). Wild bees were identified following Falk (2019) and Michez et al. (2019). In total, we collected 557 pollinator specimens belonging to 56 families via quadrat sampling, and 992 pollinator specimens belonging to 74 families via transect sampling (Table S1). We created a citizen science project on *iNaturalist* platform (inaturalist/projects) to gather further taxonomic information, for data sharing, and for public outreach.

### Data analysis

Statistical analysis was conducted in R, version 4.2.2 (R Core Team 2022).

#### How does glacier retreat affect the diversity of plant and pollinator communities?

To answer this first research question, we calculated the following variables for each replicate: (1) plant diversity (number of plant species), (2) pollinator diversity (number of pollinator species), (3) pollinator abundance (number of pollinator individuals). We also analyzed diversity using Shannon index (H), which yielded qualitatively similar results (Table S2–S3).

The impact of glacier retreat on biodiversity was tested by means of regression and mixed-effects models. Each of these three variables were used as a response variable (three separate models). For plant diversity, we fitted a generalized linear model with a Poisson distribution; glacier retreat (years, ${x}_{i}$) was the predictor as second-degree polynomial function. For pollinator diversity and pollinator abundance, we fitted a generalized linear mixed-effects model (two separate models) with Poisson distribution (Brooks et al. 2017); glacier retreat (years, ${x}_{i}$, second-degree polynomial function), plant diversity, and their statistical interaction were fixed effects; sampling method, time replicates and plots were considered as random effects. Model parameters and *p-values* were estimated with restricted maximum likelihood. The significance of fixed effects was assessed by means of Wald chi-square test using the *Anova* function (type-II) of *car* R package (Fox and Weisberg 2018).

#### How does plant diversity contribute to the change of pollinator diversity with glacier retreat?

To answer this second question, we calculated the frequency of plant species visited by pollinators as the number of flowering plant species that were visited by pollinators relative to the number of plant species flowering in each plot.

For the frequency of plant species visited by pollinators, we fitted a generalized linear mixed-effects model with a Gaussian distribution (Brooks et al. 2017); glacier retreat (years, ${x}_{i}$, second-degree polynomial function) and plant diversity were fixed effects; sampling methods, time replicates and plots were considered as random effects. Model parameters and *p-values* were estimated with restricted maximum likelihood. The significance of fixed effects was assessed by means of Wald chi-square test using the *Anova* function (type-II) of *car* R package (Fox and Weisberg 2018).

#### How does glacier retreat impact the diversity and complexity of plant–pollinator interactions?

To answer this third question, we built plant–pollinator networks for each replicate (*n* = 96) by means of bipartite networks (Bascompte and Jordano 2014). Pollination networks were constructed using the frequency of visits as a measure of relative interaction strength (Dormann et al. 2008). Such network is formed by two sets of nodes: pollinators as the upper-level group and plant species as the lower-level group, which are connected by a set of links, i.e., frequency of visits.

For each network, we calculated interaction diversity and network complexity. Interaction diversity (H) was calculated using the Shannon index (Bersier et al. 2002; Blüthgen et al. 2006) as $H=-\sum_{i=1}^{N}{p}_{i}\times {\text{log}}({p}_{i}$) where ${p}_{i}$ is the frequency of plant–pollinator interactions *i* weighted over all possible interactions *N.* This index is the weighted-mean Shannon index of interaction diversity accounting for the number of interactions in the network. Pollination network complexity was calculated using the Connectance index (Bersier et al. 2002; Dunne et al. 2002). Connectance is the proportion of realized links from the pool of all possible interactions between the species of a network as $C=\frac{interactions}{plant species \times pollinator species}$, where the denominator is network size, i.e., the number of plant species flowering multiplied by the number of pollinator species.

The impact of glacier retreat on network structure was tested by means of mixed-effects models with interaction diversity and network complexity as response variables (two separate models; Gaussian distribution); glacier retreat, plant diversity and their statistical interaction as fixed effects; sampling methods, time replicates and plots as random effects. The significance of fixed effects and parameter estimates followed the same procedure as described previously.

## Results

### Glacier retreat triggers changes in plant diversity and pollinator diversity

**Fig. 2:**
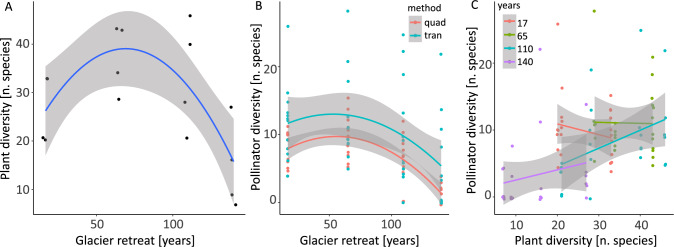
Impact of glacier retreat on plant and pollinator communities. **A** Effects of glacier retreat (*x-*axis) on plant diversity (number of species; *y*-axis). **B** Effects of glacier retreat (*x-*axis) on pollinator diversity (number of species;* y*-axis). Pollinator communities were investigated with two complementary sampling methods: quadrat (red) and transect (blue). **C** Relationship between plant diversity (number of species, *x-*axis) and pollinator diversity (number of species, *y-*axis) following glacier reatreat

Plant diversity increased after glacier retreat initially for in the first stages. But with succession proceeding, plant diversity ultimately decreased in the latest stages (beta__linear_ = -0.7 ± 0.33, *p* = 0.03, beta__quadratic_ = – 1.13 ± 0.29, *p* < 0.001, Fig. 2A, Table S2). Qualitatively similar patterns were observed for Shannon diversity and plant species flowering (Fig. S1, Tables S2).

Glacier retreat explained a significant amount of variance between pollinator communities. Pollinator diversity declined following glacier retreat (beta__linear_ = – 9.07 ± 2.1, *p* < 0.001, beta__quadratic_ = 0.21 ± 2.43, *p* = 0.93, Fig. 2B, Table S4). Qualitatively similar patterns were observed for pollinator abundance and pollinator diversity (Shannon index) (Figs. S2, Tables S3).

### Plant diversity has positive effects on pollinator diversity

Pollinator diversity increased with increasing plant diversity (beta = 0.02 ± 0.01, *p* = 0.08, Fig. 2C, Table S3). We also found that glacier retreat had negative effects on the number of plant species visited by pollinators (beta__linear_ = – 5.79 ± 1.99, *p* = 0.003, beta__quadratic_ = – 3.81 ± 2.24, *p* = 0.09, Fig. S3, Table S4). We observed a consistent pattern when considering the frequency of plant species visited by pollinators (beta__linear_ = – 0.83 ± 0.19, *p* < 0.001; beta__quadratic_ = – 0.13 ± 0.19, *p* = 0.49, Fig. S3, Table S4).

### The effects of glacier retreat on plant–pollinator interactions

**Fig. 3:**
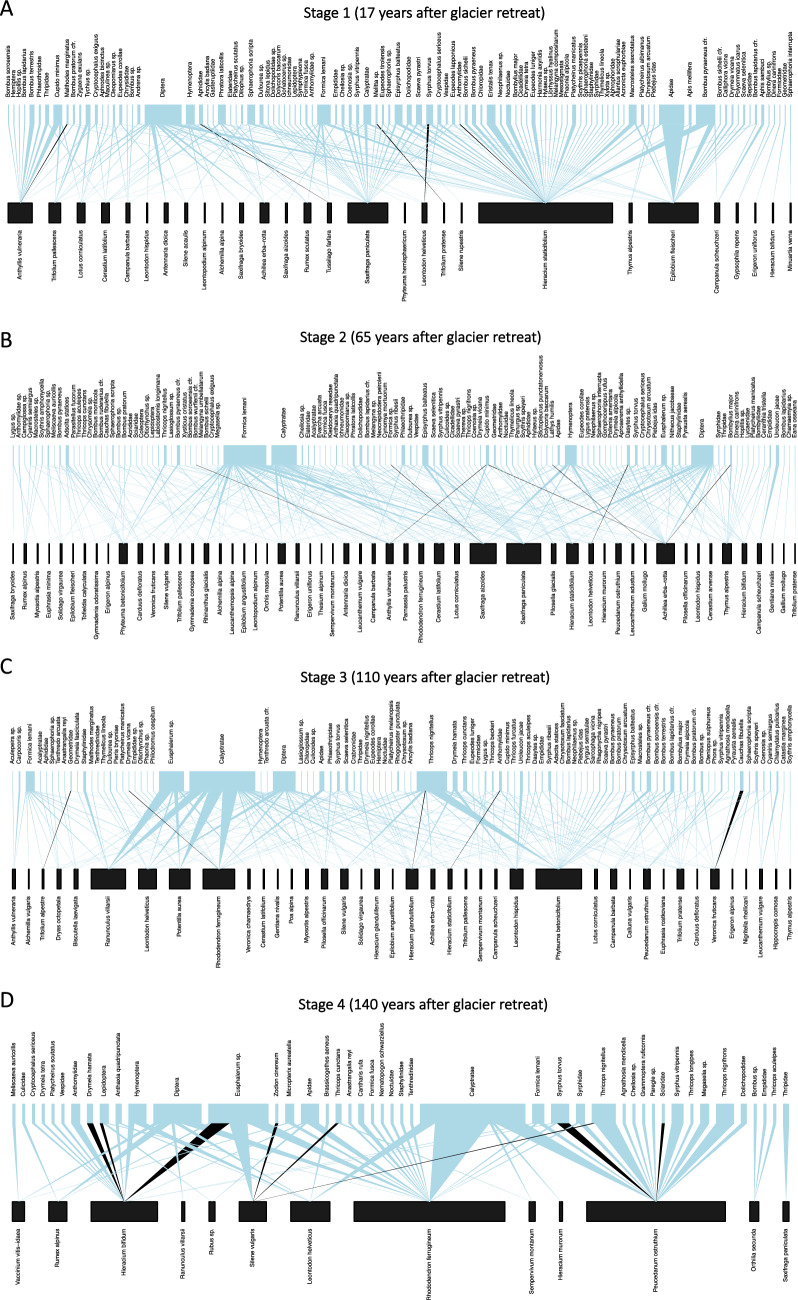
Plant–pollinator networks across 170-years glacier retreat gradient (**A**–**D** Stage 1–4). Pollinators are shown in the upper level, plants are in the lower one. Arrows represent the frequency of interactions (blue triangles for single interactions, black triangles for interaction frequency higher than 1)

We observed a total of 1549 plant–pollinator interactions. Shortly after glacier retreat, flies were the most active pollinators and *Hieracium staticifolium* was the most visited flowering plant (Fig. 3A). From 65 to 110 years after glacier retreat, flies were still the most abundant pollinator group, followed by ants (Hymenoptera: Formicidae). There, the most visited plants were quite diversified including *Epilobium fleischeri, Achillea erba-rotta, Phyteuma betonicifolium, Ranunculus villarsii, Saxifraga aizoides, Saxifraga paniculata, Cerastium arvense* (Fig. 3B, C). In the larch (*Larix decidua*) forest, 140 years after glacier retreat, flies, rove beetles (Coleoptera: Staphylinidae) and bees were the most important pollinators in the networks while *Rhododendron ferrugineum, Peucedanum ostruthium, Hieracium bifidum, Leontodon helveticus* were the most visited plant species (Fig. 3D). Flies visited diverse flowering plant species, rove beetles visited *Hieracium murorum*, *Silene vulgaris* and *Rhododendron ferrugineum* and bees were found more on *Rhododendron ferrugineum* (Fig. 3D).

**Fig. 4:**
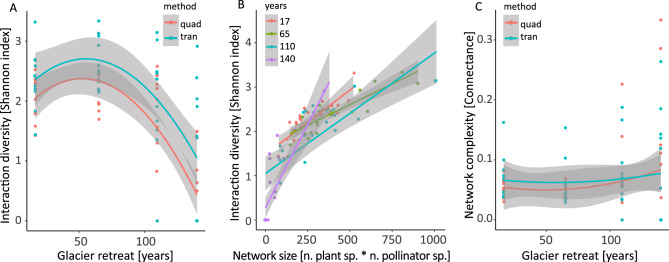
Impact of glacier retreat (*x*-axis) on **A** plant–pollinator interaction diversity (Shannon index), and **C** network complexity (Connectance). Pollinator communities were investigated with two complementary sampling methods: quadrat (red) and transect (blue). **B** Relationship between network size (number of plant species times number of pollinator species, *x-*axis) and plant–pollinator interaction diversity (Shannon index, *y-*axis) across stages of glacier retreat

We observed sharp changes in plant–pollinator interaction diversity (Shannon index) following glacier retreat (Fig. 4A). No significant differences were observed between sampling methods. Glacier retreat had negative effects on interaction diversity (beta__linear_ = – 9.09 ± 2.00, *p* < 0.001, beta__quadratic_ = 1.76 ± 2.24, *p* = 0.43, Table S5). By contrast, plant diversity had positive effects on interaction diversity (beta = 0.02 ± 0.01, *p* = 0.02, Fig. 4B, Table S5). We did not observe significant effects of glacier retreat on network complexity as connectance did not change (beta__linear_ = 0.07 ± 0.05, *p* = 0.17; beta__quadratic_ = 0.05 ± 0.05, *p* = 0.34, Fig. 4C, Table S5).

## Discussion

As global warming is rising, Alpine glaciers are predicted to lose 30–40% of their volume by 2050 and 20–60% of their surface by 2100, exposing new land and hosting novel ecosystems (Roe et al. 2017; Bosson et al 2023; Cook et al. 2023). It is therefore crucial to understand these indirect effects of climate change on species interactions to mitigate its consequences. Species interactions contribute to biodiversity maintenance and ecosystem functioning (Bascompte and Jordano 2014; Losapio et al. 2015). Although the impact of glacier retreat on biological communities is increasingly documented, species interactions remain still overlooked. Here, we address this knowledge gap by looking at the consequences of glacier retreat for networks of pollination interactions.

We found significant impacts of glacier retreat on biodiversity, plant–pollinator interactions, interaction diversity, and the way plant diversity influences pollination network. Notably, our results revealed that glacier retreat affects pollination networks via direct and indirect pathways in a two-phases process. In the first phase, glacier retreat makes space to plant colonization. This initial increase in plant diversity drives the increase in pollinator and interaction diversity. The second phase is characterized by turnover as woody species encroaches and dominates the community, decreasing the diversity of plant species in ultimate instance. The local decrease of plant diversity leads to a local decrease in pollinator and interaction diversity. The positive relationships between plant diversity and pollinator diversity observed here suggest that enhancing plant diversity can mitigate the impact of glacier retreat on pollinator communities. Our research thus can help resolve the overarching question of how to conserve ecosystems once glaciers are extinct, pointing toward a composite role of both habitat structure and biological functions.

### The importance of glaciers to support biodiversity

Glacier creates unique habitats for specialist species, adapted to cold environments and particular hydrological conditions (Gentili et al. 2020; Stibal et al. 2020; Bosson et al. 2023). Several studies have already highlighted the importance of glacier to support biodiversity, and therefore their impact when they retreat (Milner et al. 2017; Cauvy-Fraunié and Dangles 2019). Specialist species relying on glacial ecosystems are disappearing, potentially leading to species extinction (Losapio et al. 2021a). Therefore, glaciers support a specific biodiversity and their disappearance is a threat to a large number of alpine species.

We documented sharp changes in biodiversity with glacier retreat. Pioneer species with important blooming such as *Epilobium fleischeri, Hieracium staticifolium, Trifolium pallescens, Gypsophila repens, Saxifraga bryoides, Saxifraga paniculata, Saxifraga aizoides, Leucanthemopsis alpina,* and *Achillea moschata* created flower-rich habitats as early as ten years after glacier retreat. As initial glacier retreat makes space to plant colonization and can be regarded as intermediate perturbation, which are known to facilitate diversity, biodiversity increased and reached the highest peak up to *c* 60 years after glacier retreat.

However, woody encroachment triggers plant species turnover and local plant diversity decline, which ultimately decreases pollinator diversity locally. Late succession stage consists of homogenous vegetation dominated by woody species as *Larix decidua, Picea abies,* and *Rhododendron ferrugineum* which dominate the low-diversity community. As the latest stage of the succession in our study system matches the “climax” vegetation outside the glacier foreland (Delarze et al. 2015; Price et al. 2021), it is reasonable to expect that local biodiversity decline will lead to biodiversity loss at the landscape, regional scale. Even if glaciers where not retreating due to climate change, glacier fronts act as biotic filters that support specialist species, whereas woody species would not create forest communities in glacier margins (Burga et al. 2010; Lambiel et al. 2016). Taken together, these results suggest that once glaciers disappear, biodiversity would be much lower.

This trend is consistent with previous studies from polar, temperate and tropical glacier forelands that reported a sharp dynamic in plant diversity with glacier retreat (Caccianiga et al. 2006; Inouye 2020; Junker et al. 2020; Fickert 2020; Anthelme et al. 2021; Losapio et al. 2021a). Our study thus confirms ongoing global trends in biodiversity which initially increases with glacier retreat and then ultimately declines with long-term deglaciation. Notably, the decline in biodiversity we observed here may be faster than in other Alpine areas as Mont Miné glacier foreland lies in the subalpine zone: pioneer plant communities are rapidly encroached by woody vegetation and pioneer species disappear as fast as a few decades after glacier retreat. Therefore, the subalpine glacier foreland deserves particular attention.

### The impact of glacier retreat on plant–pollinator networks

The dynamics of pollinator communities matched the same trend documented for plant communities. We expected this pattern as plants are the base components of ecosystems, particularly the key food resource for pollinators. On the one hand, changes in plant communities can directly affect the composition and diversity of biological communities at higher trophic levels, and drive species interactions (Losapio et al. 2016, 2021b; Vitasse et al. 2021). On the other hand, glacier retreat on its own was less important for pollinators as compared to plant communities. Notably, we observed positive effects of plant diversity on pollination networks. The higher the richness, the higher the volume of plants that attract pollinators, the higher the pollination success (Inouye 2020).

We propose that the decline in pollinator diversity is directly driven by the decline in foraging (flower) resources, and by changes in climate or habitat only indirectly. As plant diversity has positive effects on pollinator diversity and network connectance remained constant, we suggest that pollinators can maximize the use of plant resources. This process can reflect the potential ability of pollinators to adapt to fast-changing biotic environments.

The diversity of plant–pollinator interactions increased shortly after glacier retreat, but then declined after a few decades. Changes in interaction diversity can affect community stability (Bersier et al. 2002; Dunne et al. 2002). Poorly diversified networks are the most vulnerable to species loss or perturbation by abiotic factors (Dunne et al. 2002). Conversely, increasing interaction diversity can reduce the risk of species extinction (Blüthgen et al. 2006). The highest levels of interaction diversity were observed at intermediate stages of ecosystem development, in species-rich grassland–shrubland ecotones. Increasing interaction diversity may increase functioning, suggesting that pioneer and intermediate communities are crucial for ecosystem services and require adequate protection.

With the alteration of both taxonomic and interaction diversity following glacier retreat, discussions on glacier foreland protection and management need to be opened (Zimmer et al. 2022). As shown in our case study, the development of ecosystem is extremely fast following the retreat. In less than 100 hundred years, a mature species-poor forest has overtaken diverse plant and insect communities, decreasing biodiversity locally. In Switzerland, as for most of temperate regions, forests are expanding, especially in mountain area (Pretzsch et al. 2014, 2023). When forests expand, they also take space over various important habitats for biodiversity, such as alpine grasslands and glacier moraines, which are threatened in Switzerland (Klaus et al. 2023). Alpine grasslands are the richest habitat in Switzerland and in temperate Europe and host numerous plant and pollinators (Schils et al. 2022). As shown in our results, pioneer grasslands are hosting the highest interaction diversity. Although late forest provides various ecosystem services, such as wood production or protection against natural hazards, it is important to understand such nuances and address the trade-offs among diverse sets of ecosystem services. For instance, our study reports a sharp decline in pollinators and interaction diversity in forests.

We are reaching a point where it is necessary to take actions to manage glacier foreland in order to protect the unique biodiversity close to glacier margins and promoting alpine grasslands to enhanced species interactions, ecological networks, and ecosystem functioning. Notably, there is a crucial difference between protecting forests and limiting forest encroachment. As much as it is important to maintain current forests, it is equally key to support diverse communities and diversified landscapes that also include open habitats such grasslands and shrublands.

In conclusion, our results confirm the hypothesis that initial glacier retreat can make space to support plant and pollinator diversity, but in the long term it will turn into negative effects which decrease biodiversity locally. Plant diversity plays an importance role in driving pollinators and supporting the stability of pollination networks. Increasing plant diversity would help to maintain the diversity of pollinators and build up robust networks. Enhancing the diversity of plants may therefore be a key strategy for halting the erosion of ecological networks under the negative impacts of global warming while increasing ecosystem functioning.

## Supplementary Information

Below is the link to the electronic supplementary material.

Supplementary file1 (396 723 KB)

## Data Availability

All datasets on which the conclusions of the paper rely are publicly available to readers on *Zenodo* (10.5281/zenodo.10911864).

## Code availability

The R script (no novel code) to reproduce the analyses and figures is deposited on GitHub (https://github.com/losapio/AlpineBotany2024).
