# Supplementary material for: Glacier retreat triggers changes in biodiversity and plant–pollinator interaction diversity
Source: Alp Bot. 2024 Apr 9;134(2):171–82. doi: 10.1007/s00035-024-00309-9 (PMC11607045; doi:10.1007/s00035-024-00309-9)
Supplement: Supplementary file 1 — Supplementary file1 (396 723 KB) [file 35_2024_309_MOESM1_ESM.docx]

Alpine Botany

APPENDIX

Glacier retreat triggers changes in biodiversity and plant–pollinator interaction diversity

Bao Ngan Tu, Nora Khelidj, Pierfilippo Cerretti, Natasha de Vere, Andrea Ferrari, Francesco Paone, Carlo Polidori, Jürg Schmid, Daniele Sommaggio, Gianalberto Losapio

**Table S1.** Summary table index of plant communities, pollinator communities and their interaction networks. Plot: 4 plots (A, B, C, D) per each stage (1, 2, 3, 4). Glacier retreat (years). Sampling method: quad = quadrat and tran = transect. Time replicate (3 random replicates for each sampling methods). Number of plant species was counted within each plot and 1m buffer. Pollinator abundance: n = number of individual pollinators. Plant–pollinator interactions were studied by sampling flower visitors (pollinators) on plants. Plant diversity, pollinator diversity, interaction diversity (Shannon index - H): $H= - \sum_{i=1}^{n} p_{i}lnp_{i}$. Connectance: C = links/species^2^.

| Plot | Glacier retreat (years) | Method | Time replicate | Number of plant species | Plant divresity (Shannon) | Pollinator abundance | Number of pollinator species | Pollinator diversity (Shannon) | Interaction diversity (Shannon) | Connectance |
| --- | --- | --- | --- | --- | --- | --- | --- | --- | --- | --- |
| 1A | 17 | quad | 1 | 20 | 2.99 | 13 | 10 | 2.13 | 2.35 | 0.07 |
| 1A | 17 | quad | 2 | 20 | 2.99 | 10 | 10 | 2.30 | 2.30 | 0.05 |
| 1A | 17 | quad | 3 | 20 | 2.99 | 13 | 9 | 2.03 | 2.20 | 0.07 |
| 1B | 17 | quad | 1 | 21 | 3.04 | 10 | 9 | 2.16 | 2.16 | 0.05 |
| 1B | 17 | quad | 2 | 21 | 3.04 | 15 | 11 | 2.33 | 2.52 | 0.06 |
| 1B | 17 | quad | 3 | 21 | 3.04 | 17 | 10 | 2.11 | 2.26 | 0.08 |
| 1C | 17 | quad | 1 | 33 | 3.49 | 9 | 4 | 1.14 | 1.42 | 0.06 |
| 1C | 17 | quad | 2 | 33 | 3.49 | 6 | 5 | 1.56 | 1.79 | 0.03 |
| 1C | 17 | quad | 3 | 33 | 3.49 | 6 | 6 | 1.79 | 1.79 | 0.03 |
| 1D | 17 | quad | 1 | 33 | 3.48 | 10 | 5 | 1.47 | 2.02 | 0.06 |
| 1D | 17 | quad | 2 | 33 | 3.48 | 12 | 10 | 2.21 | 2.36 | 0.03 |
| 1D | 17 | quad | 3 | 33 | 3.48 | 9 | 6 | 1.58 | 1.58 | 0.04 |
| 2A | 65 | quad | 1 | 29 | 3.35 | 17 | 11 | 2.11 | 2.58 | 0.05 |
| 2A | 65 | quad | 2 | 29 | 3.35 | 19 | 11 | 2.21 | 2.45 | 0.06 |
| 2A | 65 | quad | 3 | 29 | 3.35 | 10 | 5 | 1.50 | 1.69 | 0.07 |
| 2B | 65 | quad | 1 | 43 | 3.76 | 19 | 7 | 1.30 | 1.95 | 0.06 |
| 2B | 65 | quad | 2 | 43 | 3.76 | 25 | 14 | 2.37 | 2.45 | 0.04 |
| 2B | 65 | quad | 3 | 43 | 3.76 | 17 | 9 | 2.06 | 2.36 | 0.04 |
| 2C | 65 | quad | 1 | 43 | 3.76 | 37 | 15 | 2.23 | 3.13 | 0.06 |
| 2C | 65 | quad | 2 | 43 | 3.76 | 22 | 12 | 2.10 | 2.43 | 0.04 |
| 2C | 65 | quad | 3 | 43 | 3.76 | 10 | 6 | 1.49 | 1.83 | 0.04 |
| 2D | 65 | quad | 1 | 34 | 3.52 | 15 | 10 | 2.07 | 2.48 | 0.04 |
| 2D | 65 | quad | 2 | 34 | 3.52 | 10 | 8 | 1.97 | 1.97 | 0.04 |
| 2D | 65 | quad | 3 | 34 | 3.52 | 7 | 7 | 1.94 | 1.94 | 0.03 |
| 3A | 110 | quad | 1 | 40 | 3.67 | 19 | 7 | 1.64 | 2.57 | 0.07 |
| 3A | 110 | quad | 2 | 40 | 3.67 | 25 | 9 | 1.92 | 2.20 | 0.07 |
| 3A | 110 | quad | 3 | 40 | 3.67 | 17 | 7 | 1.57 | 1.90 | 0.06 |
| 3B | 110 | quad | 1 | 21 | 3.04 | 19 | 4 | 0.82 | 0.82 | 0.23 |
| 3B | 110 | quad | 2 | 21 | 3.04 | 6 | 5 | 1.56 | 1.56 | 0.06 |
| 3B | 110 | quad | 3 | 21 | 3.04 | 0 | 0 | 0 | 0 | 0 |
| 3C | 110 | quad | 1 | 28 | 3.31 | 16 | 10 | 1.92 | 2.42 | 0.06 |
| 3C | 110 | quad | 2 | 28 | 3.31 | 15 | 8 | 1.87 | 2.43 | 0.07 |
| 3C | 110 | quad | 3 | 28 | 3.31 | 0 | 0 | 0 | 0 | 0 |
| 3D | 110 | quad | 1 | 46 | 3.82 | 32 | 5 | 0.55 | 1.29 | 0.14 |
| 3D | 110 | quad | 2 | 46 | 3.82 | 24 | 12 | 2.16 | 2.27 | 0.04 |
| 3D | 110 | quad | 3 | 46 | 3.82 | 12 | 9 | 2.02 | 2.02 | 0.03 |
| 4A | 140 | quad | 1 | 7 | 1.94 | 8 | 4 | 1.32 | 1.49 | 0.29 |
| 4A | 140 | quad | 2 | 7 | 1.94 | 0 | 0 | 0 | 0 | 0 |
| 4A | 140 | quad | 3 | 7 | 1.94 | 0 | 0 | 0 | 0 | 0 |
| 4B | 140 | quad | 1 | 9 | 2.19 | 9 | 3 | 0.84 | 0.84 | 0.33 |
| 4B | 140 | quad | 2 | 9 | 2.19 | 0 | 0 | 0 | 0 | 0 |
| 4B | 140 | quad | 3 | 9 | 2.19 | 0 | 0 | 0 | 0 | 0 |
| 4C | 140 | quad | 1 | 16 | 2.75 | 2 | 1 | 0 | 0 | 0.13 |
| 4C | 140 | quad | 2 | 16 | 2.75 | 0 | 0 | 0 | 0 | 0 |
| 4C | 140 | quad | 3 | 16 | 2.75 | 0 | 0 | 0 | 0 | 0 |
| 4D | 140 | quad | 1 | 27 | 3.29 | 6 | 2 | 0.63 | 0.63 | 0.11 |
| 4D | 140 | quad | 2 | 27 | 3.29 | 4 | 4 | 1.38 | 1.38 | 0.04 |
| 4D | 140 | quad | 3 | 27 | 3.29 | 5 | 2 | 0.50 | 0.50 | 0.09 |
| 1A | 17 | tran | 1 |  |  | 14 | 7 | 1.67 | 2.20 | 0.1 |
| 1A | 17 | tran | 2 |  |  | 35 | 26 | 3.08 | 3.31 | 0.07 |
| 1A | 17 | tran | 3 |  |  | 13 | 4 | 1.26 | 1.43 | 0.16 |
| 1B | 17 | tran | 1 |  |  | 9 | 6 | 1.67 | 1.83 | 0.07 |
| 1B | 17 | tran | 2 |  |  | 25 | 16 | 2.67 | 2.67 | 0.07 |
| 1B | 17 | tran | 3 |  |  | 21 | 12 | 2.24 | 2.42 | 0.08 |
| 1C | 17 | tran | 1 |  |  | 17 | 13 | 2.50 | 2.58 | 0.04 |
| 1C | 17 | tran | 2 |  |  | 12 | 9 | 2.13 | 2.25 | 0.04 |
| 1C | 17 | tran | 3 |  |  | 20 | 13 | 2.41 | 2.41 | 0.05 |
| 1D | 17 | tran | 1 |  |  | 10 | 8 | 2.02 | 2.16 | 0.04 |
| 1D | 17 | tran | 2 |  |  | 20 | 15 | 2.62 | 2.69 | 0.04 |
| 1D | 17 | tran | 3 |  |  | 18 | 12 | 2.39 | 2.58 | 0.05 |
| 2A | 65 | tran | 1 |  |  | 10 | 9 | 2.16 | 2.16 | 0.04 |
| 2A | 65 | tran | 2 |  |  | 36 | 28 | 3.22 | 3.33 | 0.04 |
| 2A | 65 | tran | 3 |  |  | 20 | 11 | 2.20 | 2.59 | 0.06 |
| 2B | 65 | tran | 1 |  |  | 13 | 9 | 2.03 | 2.45 | 0.03 |
| 2B | 65 | tran | 2 |  |  | 43 | 21 | 2.74 | 3.08 | 0.05 |
| 2B | 65 | tran | 3 |  |  | 31 | 7 | 1.48 | 2.34 | 0.1 |
| 2C | 65 | tran | 1 |  |  | 33 | 5 | 1.20 | 2.32 | 0.15 |
| 2C | 65 | tran | 2 |  |  | 35 | 18 | 2.46 | 3.12 | 0.05 |
| 2C | 65 | tran | 3 |  |  | 32 | 13 | 2.15 | 2.92 | 0.06 |
| 2D | 65 | tran | 1 |  |  | 16 | 11 | 2.18 | 2.51 | 0.04 |
| 2D | 65 | tran | 2 |  |  | 9 | 9 | 2.19 | 2.19 | 0.03 |
| 2D | 65 | tran | 3 |  |  | 11 | 9 | 2.14 | 2.14 | 0.04 |
| 3A | 110 | tran | 1 |  |  | 45 | 6 | 1.16 | 2.46 | 0.19 |
| 3A | 110 | tran | 2 |  |  | 72 | 25 | 2.47 | 3.14 | 0.07 |
| 3A | 110 | tran | 3 |  |  | 25 | 10 | 1.60 | 2.49 | 0.06 |
| 3B | 110 | tran | 1 |  |  | 14 | 4 | 1.05 | 1.35 | 0.17 |
| 3B | 110 | tran | 2 |  |  | 8 | 4 | 1.21 | 1.66 | 0.1 |
| 3B | 110 | tran | 3 |  |  | 0 | 0 | 0 | 0 | 0 |
| 3C | 110 | tran | 1 |  |  | 20 | 13 | 2.48 | 2.48 | 0.05 |
| 3C | 110 | tran | 2 |  |  | 35 | 19 | 2.60 | 3.02 | 0.07 |
| 3C | 110 | tran | 3 |  |  | 13 | 6 | 1.28 | 1.51 | 0.08 |
| 3D | 110 | tran | 1 |  |  | 18 | 9 | 1.71 | 2.36 | 0.04 |
| 3D | 110 | tran | 2 |  |  | 34 | 22 | 2.90 | 3.14 | 0.03 |
| 3D | 110 | tran | 3 |  |  | 8 | 5 | 1.55 | 1.90 | 0.03 |
| 4A | 140 | tran | 1 |  |  | 4 | 4 | 1.38 | 1.38 | 0.14 |
| 4A | 140 | tran | 2 |  |  | 0 | 0 | 0 | 0 | 0 |
| 4A | 140 | tran | 3 |  |  | 0 | 0 | 0 | 0 | 0 |
| 4B | 140 | tran | 1 |  |  | 19 | 8 | 1.78 | 1.90 | 0.26 |
| 4B | 140 | tran | 2 |  |  | 0 | 0 | 0 | 0 | 0 |
| 4B | 140 | tran | 3 |  |  | 0 | 0 | 0 | 0 | 0 |
| 4C | 140 | tran | 1 |  |  | 21 | 11 | 1.88 | 2.03 | 0.12 |
| 4C | 140 | tran | 2 |  |  | 48 | 22 | 2.83 | 1.91 | 0.14 |
| 4C | 140 | tran | 3 |  |  | 0 | 0 | 0 | 0 | 0 |
| 4D | 140 | tran | 1 |  |  | 15 | 3 | 0.86 | 1.41 | 0.19 |
| 4D | 140 | tran | 2 |  |  | 20 | 14 | 2.38 | 2.38 | 0.05 |
| 4D | 140 | tran | 3 |  |  | 0 | 0 | 0 | 0 | 0 |

**Table S2.** Print model output and effect size for the effects of glacier retreat on plant diversity (number of species), plant diversity (Shannon index – H), number of plant species flowering.

| Plant diversity (number of species) |
| --- |
| ## Family: genpois ( log )  ## Formula: plant_richness ~ poly(years,2)  ## Data: pool_df  ##  ## AIC BIC logLik deviance df.resid  ## 120.1 123.2 -56.1 112.1 12  #  ## Dispersion parameter for genpois family (): 2.63  ##  ## Conditional model:  ## Estimate Std. Error z value Pr(>\|z\|)   \| ## \| (Intercept) \| 3.28217 \| 0.08041 \| 40.82 \| < 2e-16 \| *** \| \| --- \| --- \| --- \| --- \| --- \| --- \| --- \| \| ## \| poly(years, 2)1 \| -0.71860 \| 0.33787 \| -2.13 \| 0.033431 \| * \|   ## poly(years, 2)2 -1.14975 0.30260 -3.80 0.000145 ***  ## ---  ## Signif. codes: 0 '***’ 0.001 '**’ 0.01 '*' 0.05 '. ' 0.1 ' ' 1  ## Analysis of Deviance Table (Type II Wald chisquare tests)  ##  ## Response: plant_richness  ## Chisq Df Pr(>Chisq)  ## poly(years, 2) 17.775 2 0.0001381 ***  ## ---  ## Signif. codes: 0 '***' 0.001 '**' 0.01 '*' 0.05 '. ' 0.1 ' ' 1  ## For one-way between subjects designs, partial eta squared is equivalent  ## to eta squared. Returning eta squared.  ## # Effect Size for ANOVA  ##  ## Parameter \| Cohen’s f \| 95% CI  ##  ## poly(years, 2) \| 1.09 \| [0.43, Inf]  ##  ## - One-sided CIs: upper bound fixed at [Inf]. |
| Plant diversity (Shannon index – H) |
| ## Call:  ## lm(formula = plant_diversity ~ poly(years, 2), data = pool_df)  ##  ## Residuals:  ## Min 1Q Median 3Q Max  ## -0.6641 -0.2422 0.0212 0.2644 0.6858  ##  ## Coefficients:  ## Estimate Std. Error t value Pr(>\|t\|)  ## (Intercept) 3.21807 0.09677 33.254 5.79e-14 ***  ## poly(years, 2)1 -0.87871 0.38709 -2.270 0.04087 *  ## poly(years, 2)2 -1.31255 0.38709 -3.391 0.00483 **  ## ---  ## Signif. codes: 0 '***' 0.001 '**' 0.01 '*' 0.05 '.' 0.1 ' ' 1  ##  ## Residual standard error: 0.3871 on 13 degrees of freedom  ## Multiple R-squared: 0.5616, Adjusted R-squared: 0.4941  ## F-statistic: 8.325 on 2 and 13 DF, p-value: 0.004703  ## Anova Table (Type II tests)  ##  ## Response: plant_diversity  ## Sum Sq Df F value Pr(>F)  ## poly(years, 2) 2.4949 2 8.3253 0.004703 **  ## Residuals 1.9479 13  ## ---  ## Signif. codes: 0 '***' 0.001 '**' 0.01 '*' 0.05 '.' 0.1 ' ' 1  ## For one-way between subjects designs, partial eta squared is equivalent  ## to eta squared. Returning eta squared.  ## # Effect Size for ANOVA  ##  ## Parameter \| Cohen's f \| 95% CI  ## ----------------------------------------  ## poly(years, 2) \| 1.13 \| [0.47, Inf]  ##  ## - One-sided CIs: upper bound fixed at [Inf]. |
| Number of plant species flowering |
| ## Family: nbinom2 ( log )  ## Formula: n.plsp.flw ~ poly(years, 2) + (1 \| replicate) +(1\| plot)  ## Data: summaryt_quad  ##  ## AIC BIC logLik deviance df.resid  ## 285.4 296.6 -136.7 273.4 42  ##  ## Random effects:  ##  ## Conditional model:  ## Groups Name Variance Std.Dev.  ## replicate (Intercept) 0.05427 0.2330  ## plot (Intercept) 0.06360 0.2522  ## Number of obs: 48, groups: replicate, 3; plot, 4  ##  ## Dispersion parameter for nbinom2 family (): 11  ##  ## Conditional model:  ## Estimate Std. Error z value Pr(>\|z\|)  ## (Intercept) 2.0765 0.1975 10.514 < 2e-16***  ## poly(years, 2)1 -1.8475 0.5101 -3.622 0.000292***  ## poly(years, 2)2 -3.0114 0.4729 -6.368 1.91e-10***  ## ---  ## Signif. codes: 0 '***' 0.001 '**' 0.01 '*' 0.05 '. '0.1 ' ' 1  ## Analysis of Deviance Table (Type II Wald chisquare tests)  ##  ## Response: n.plsp.flw  ## Chisq Df Pr(>Chisq)  ## poly(years, 2) 48.361 2 3.152e-11 ***  ## ---  ## Signif. codes: 0 '***' 0.001 '**' 0.01 '*' 0.05 '.' 0.1 ' ' 1  ## # Effect Size for ANOVA (Type I)  ##  ## Parameter \| Cohen's f (partial) \| 95% CI  ##  ## poly(years, 2) \| 0.93 \| [0.60, Inf]  ## replicate \| 0.03 \| [0.00, Inf]  ## plot \| 0.48 \| [0.09, Inf]  ##  ## - One-sided CIs: upper bound fixed at [Inf]. |

**Table S3.** Print model output and effect size for the effects of glacier retreat, plant diversity as predictors, sampling method, time replicate and plots as random effects on pollinator diversity (number of species), pollinator abundance (number of individual), pollinator diversity (Shannon index – H).

| Pollinator diversity (number of species) |
| --- |
| ## Family: nbinom2 ( log )  ## Formula:  ## insect_richness ~ poly(years, 2) * plant_richness + (1 \| plot/replicate2)  ## +(1 \| method)  ## Data: summaryt  ##  ## AIC BIC logLik deviance df.resid  ## 570.3 595.9 -275.1 550.3 86  ##  ## Random effects:  ##  ## Conditional model:  ## Groups Name Variance Std.Dev.  ## replicate2:plot (Intercept) 9.852e-02 3.139e-01  ## plot (Intercept) 8.005e-10 2.829e-05  ## method (Intercept) 4.188e-02 2.046e-01  ## Number of obs: 96, groups: replicate2:plot, 24; plot, 4; method, 2  ##  ## Dispersion parameter for nbinom2 family (): 5.32  ##  ## Conditional model:  ## Estimate Std. Error z value Pr(>\|z\|)  ## (Intercept) 1.474543 0.312236 4.723 2.33e-06 ***  ## poly(years, 2)1 -9.065278 2.107187 -4.302 1.69e-05 ***  ## poly(years, 2)2 0.212956 2.433863 0.087 0.93028  ## plant_richness 0.016172 0.009084 1.780 0.07504 .  ## poly(years, 2)1:plant_richness 0.218401 0.076099 2.870 0.00411 **  ## poly(years, 2)2:plant_richness -0.052050 0.080758 -0.645 0.51924  ## ---  ## Signif. codes: 0 '***' 0.001 '**' 0.01 '*' 0.05 '.' 0.1 ' ' 1  ## Analysis of Deviance Table (Type II Wald chisquare tests)  ##  ## Response: insect_richness  ## Chisq Df Pr(>Chisq)  ## poly(years, 2) 24.4987 2 4.788e-06 ***  ## plant_richness 7.2617 1 0.007044 **  ## poly(years, 2):plant_richness 8.2460 2 0.016196 *  ## ---  ## Signif. codes: 0 '***' 0.001 '**' 0.01 '*' 0.05 '.' 0.1 ' ' 1  ## # Effect Size for ANOVA (Type I)  ##  ## Parameter \| Cohen's f (partial) \| 95% CI  ## -----------------------------------------------------------------  ## poly(years, 2) \| 0.85 \| [0.60, Inf]  ## plant_richness \| 0.27 \| [0.06, Inf]  ## plot \| 0.26 \| [0.00, Inf]  ## method \| 0.52 \| [0.31, Inf]  ## poly(years, 2):plant_richness \| 0.29 \| [0.00, Inf]  ## plot:replicate2 \| 0.95 \| [0.51, Inf]  ##  ## - One-sided CIs: upper bound fixed at [Inf]. |
| Pollinator abundance (number of individual) |
| ## Family: nbinom2 ( log )  ## Formula: insect_abundance ~ poly(years, 2) * plant_richness + (1 \| replicate2) + (1 \| method) + (1 \| plot)  ## Data: summary  ##  ## AIC BIC logLik deviance df.resid  ## 696.3 722.0 -338.2 676.3 86  ##  ## Random effects:  ##  ## Conditional model:  ## Groups Name Variance Std.Dev.  ## replicate2 (Intercept) 0.158914 0.39864  ## method (Intercept) 0.026133 0.16166  ## plot (Intercept) 0.009812 0.09905  ## Number of obs: 96, groups: replicate2, 6; method, 2; plot, 4  ##  ## Dispersion parameter for nbinom2 family (): 2.48  ##  ## Conditional model:  ## Estimate Std. Error z value Pr(>\|z\|)  ##(Intercept) 1.67997 0.39922 4.208 2.58e-05***  ## poly(years, 2)1 -8.72460 2.53280 -3.445 0.000572***  ## poly(years, 2)2 2.20873 2.88603 0.765 0.444083  ## plant_richness 0.03007 0.01179 2.551 0.010729*  ## poly(years, 2)1:plant_richness 0.26421 0.09271 2.850 0.004374**  ## poly(years, 2)2:plant_richness -0.10594 0.09757 -1.086 0.277613  ## ---  ## Signif. codes: 0 '***' 0.001 '**' 0.01 '*' 0.05 '.' 0.1 ' ' 1  ## Analysis of Deviance Table (Type II Wald chisquare tests)  ##  ## Response: insect_abundance  ## Chisq Df Pr(>Chisq)  ## poly(years, 2) 4.9151 2 0.0856438 .  ## plant_richness 11.8155 1 0.0005874 ***  ## poly(years, 2):plant_richness 8.2858 2 0.0158768 *  ## ---  ## Signif. codes: 0 '***' 0.001 '**' 0.01 '*' 0.05 '.' 0.1 ' ' 1  ## Effect Size for ANOVA (Type I)  ##  ## Parameter \| Cohen's f (partial) \| 95% CI  ## --------------------------------------------------------------------  ##poly(years, 2) \| 0.68 \| [0.47, Inf]  ##plant_richness \| 0.46 \| [0.27, Inf]  ##replicate2 \| 0.75 \| [0.50, Inf]  ##plot \| 0.50 \| [0.27, Inf]  ##poly(years, 2):plant_richness \| 0.25 \| [0.00, Inf]  ##  ##- One-sided CIs: upper bound fixed at [Inf]. |
| Pollinator diversity (Shannon index – H) |
| ## Linear mixed model fit by REML. t-tests use Satterthwaite's method [  ## lmerModLmerTest]  ## Formula:  ## insect_diversity ~ poly(years, 2) * plant_richness + (1 \| replicate2) +  ## (1 \| method) + (1 \| plot)  ## Data: summaryt  ##  ## REML criterion at convergence: 185.7  ##  ## Scaled residuals:  ## Min 1Q Median 3Q Max  ## -2.0426 -0.6366 0.1252 0.5640 2.8440  ##  ## Random effects:  ## Groups Name Variance Std.Dev.  ## replicate2 (Intercept) 1.133e-01 3.365e-01  ## plot (Intercept) 3.982e-11 6.310e-06  ## method (Intercept) 1.901e-02 1.379e-01  ## Residual 3.254e-01 5.704e-01  ## Number of obs: 96, groups: replicate2, 6; plot, 4; method, 2  ##  ## Fixed effects:  ## Estimate Std. Error df t value Pr(>\|t\|)  ## (Intercept) 1.276582 0.295611 7.301870 4.318 0.00316  ## poly(years, 2)1 -8.267684 1.951038 84.999979 -4.238 5.71e-05  ## poly(years, 2)2 0.141546 2.260176 84.999982 0.063 0.95021  ## plant_richness 0.011297 0.007958 84.999962 1.419 0.15942  ## poly(years, 2)1:plant_richness 0.150943 0.071894 84.999980 2.100 0.03874  ## poly(years, 2)2:plant_richness -0.048080 0.073072 84.999981 -0.658 0.51233  ##  ## (Intercept) **  ## poly(years, 2)1 ***  ## poly(years, 2)2  ## plant_richness  ## poly(years, 2)1:plant_richness *  ## poly(years, 2)2:plant_richness  ## ---  ## Signif. codes: 0 '***' 0.001 '**' 0.01 '*' 0.05 '.' 0.1 ' ' 1  ##  ## Correlation of Fixed Effects:  ## (Intr) pl(,2)1 pl(,2)2 plnt_r p(,2)1:  ## ply(yrs,2)1 -0.349  ## ply(yrs,2)2 -0.281 -0.331  ## plnt_rchnss -0.776 0.444 0.168  ## ply(y,2)1:_ 0.292 -0.940 0.282 -0.339  ## ply(y,2)2:_ 0.117 0.379 -0.935 0.065 -0.289  ## optimizer (nloptwrap) convergence code: 0 (OK)  ## boundary (singular) fit: see help('isSingular')  ## Analysis of Deviance Table (Type II Wald chisquare tests)  ##  ## Response: insect_diversity  ## Chisq Df Pr(>Chisq)  ## poly(years, 2) 49.0112 2 2.277e-11 ***  ## plant_richness 5.1241 1 0.0236 *  ## poly(years, 2):plant_richness 4.4109 2 0.1102  ## ---  ## Signif. codes: 0 '***' 0.001 '**' 0.01 '*' 0.05 '.' 0.1 ' ' 1  ## Effect Size for ANOVA (Type I)  ##  ## Parameter \| Cohen's f (partial) \| 95% CI  ## ------------------------------------------------------------------  ## poly(years, 2) \| 0.68 \| [0.47, Inf]  ## plant_richness \| 0.46 \| [0.27, Inf]  ## replicate2 \| 0.75 \| [0.50, Inf]  ## plot \| 0.50 \| [0.27, Inf]  ## poly(years, 2):plant_richness \| 0.25 \| [0.00, Inf]  ##  ## - One-sided CIs: upper bound fixed at [Inf]. |

**Table S4.** Print model output and effect size for the effects of glacier retreat, plant diversity as predictors, sampling method, time replicate and plots as random effects on the number of plant species visited by pollinators, the frequency of plant species visited by pollinators.

| Number of plant species visited by pollinators |
| --- |
| ## Generalized linear mixed model fit by maximum likelihood (Laplace Approximation) ## ['glmerMod']  ## Family: poisson ( log )  ## Formula: summaryt$n.plsp.vis ~ poly(years, 2) * plant_richness + (1 \|  ## replicate2) + (1 \| method) + (1 \| plot)  ## Data: summaryt  ##  ## AIC BIC logLik deviance df.resid  ## 380.3 403.4 -181.2 362.3 87  ##  ## Scaled residuals:  ## Min 1Q Median 3Q Max  ## -1.5416 -0.6423 -0.1458 0.4917 2.8817  ##  ## Random effects:  ## Groups Name Variance Std.Dev.  ## replicate2 (Intercept) 7.797e-02 0.2792313  ## plot (Intercept) 2.074e-03 0.0455394  ## method (Intercept) 4.683e-08 0.0002164  ## Number of obs: 96, groups: replicate2, 6; plot, 4; method, 2  ##  ## Fixed effects:  ## Estimate Std. Error z value Pr(>\|z\|)  ## (Intercept) 0.328171 0.255254 1.286 0.19856  ## poly(years, 2)1 -5.794002 1.992004 -2.909 0.00363 **  ## poly(years, 2)2 -3.809398 2.242665 -1.699 0.08939 .  ## plant_richness 0.032614 0.007349 4.438 9.07e-06 ***  ## poly(years, 2)1:plant_richness 0.122310 0.067000 1.826 0.06792 .  ## poly(years, 2)2:plant_richness 0.069423 0.071337 0.973 0.33046  ## ---  ## Signif. codes: 0 ‘***’ 0.001 ‘**’ 0.01 ‘*’ 0.05 ‘.’ 0.1 ‘ ’ 1  ##  ## Correlation of Fixed Effects:  ## (Intr) pl(,2)1 pl(,2)2 plnt_r p(,2)1:  ## ply(yrs,2)1 -0.187  ## ply(yrs,2)2 -0.076 -0.072  ## plnt_rchnss -0.839 0.249 -0.080  ## ply(y,2)1:_ 0.178 -0.945 0.057 -0.192  ## ply(y,2)2:_ -0.069 0.139 -0.948 0.283 -0.083  ## Analysis of Deviance Table (Type II Wald chisquare tests)  ##  ## Response: summaryt$n.plsp.vis  ## Chisq Df Pr(>Chisq)  ## poly(years, 2) 15.9521 2 0.0003436 ***  ## plant_richness 22.5859 1 2.01e-06 ***  ## poly(years, 2):plant_richness 4.6067 2 0.0999227 .  ## ---  ## Signif. codes: 0 ‘***’ 0.001 ‘**’ 0.01 ‘*’ 0.05 ‘.’ 0.1 ‘ ’ 1 |
| The frequency of plant species visited by pollinators |
| ## Linear mixed model fit by REML. t-tests use Satterthwaite's method  ## ['lmerModLmerTest']  ## Formula: std.plvis.plflw ~ poly(years, 2) + (1 \| replicate) + (1 \| plot)  ## Data: summaryt_quad  ##  ## REML criterion at convergence: -11.6  ##  ## Scaled residuals:  ## Min 1Q Median 3Q Max  ## -2.2304 -0.6649 -0.3065 0.5032 2.4999  ##  ## Random effects:  ## Groups Name Variance Std.Dev.  ## plot (Intercept) 0.0002413 0.01554  ## replicate (Intercept) 0.0357233 0.18901  ## Residual 0.0364077 0.19081  ## Number of obs: 48, groups: plot, 4; replicate, 3  ##  ## Fixed effects:  ## Estimate Std. Error df t value Pr(>\|t\|)  ## (Intercept) 0.3678 0.1128 2.0132 3.260 0.0819 .  ## poly(years, 2)1 -0.8323 0.1908 39.9999 -4.362 8.81e-05 ***  ## poly(years, 2)2 -0.1344 0.1908 39.9999 -0.704 0.4853  ## ---  ## Signif. codes: 0 ‘***’ 0.001 ‘**’ 0.01 ‘*’ 0.05 ‘.’ 0.1 ‘ ’ 1  ##  ## Correlation of Fixed Effects:  ## (Intr) p(,2)1  ## ply(yrs,2)1 0.000  ## ply(yrs,2)2 0.000 0.000  ## Analysis of Deviance Table (Type II Wald chisquare tests)  ##  ## Response: std.plvis.plflw  ## Chisq Df Pr(>Chisq)  ## poly(years, 2) 19.522 2 5.765e-05 ***  ## ---  ## Signif. codes: 0 ‘***’ 0.001 ‘**’ 0.01 ‘*’ 0.05 ‘.’ 0.1 ‘ ’ 1 |

**Table S5**. Print model output and effect size for the effects of glacier retreat, plant diversity as predictors, sampling method, time replicates and plots as random effects on plant–pollinator interaction diversity (Shannon index) and network complexity (Connectance).

| Plant–pollinator interaction diversity (Shannon index) |
| --- |
| ## Linear mixed model fit by REML. t-tests use Satterthwaite's method  ## ['lmerModLmerTest']  ## Formula: intdiv ~ poly(years, 2) * plant_richness + (1 \| replicate2) + (1 ## \| method) + (1 \| plot)  ## Data: summaryt_int  ##  ## REML criterion at convergence: 185  ##  ## Scaled residuals:  ## Min 1Q Median 3Q Max  ## -2.17127 -0.57221 -0.01432 0.62377 2.83184  ##  ## Random effects:  ## Groups Name Variance Std.Dev.  ## replicate2 (Intercept) 0.12318 0.3510  ## plot (Intercept) 0.01166 0.1080  ## method (Intercept) 0.04725 0.2174  ## Residual 0.31404 0.5604  ## Number of obs: 96, groups: replicate2, 6; plot, 4; method, 2  ##  ## Fixed effects:  ## Estimate Std. Error df t value Pr(>\|t\|)  ## (Intercept) 1.160470 0.337055 5.340632 3.443 0.0165 *  ## poly(years, 2)1 -9.091575 2.001865 70.752967 -4.542 2.24e-05 ***  ## poly(years, 2)2 1.756223 2.238329 84.815266 0.785 0.4349  ## plant_richness 0.022345 0.008699 29.740722 2.569 0.0155 *  ## poly(years,2)1:plant_richness 0.183461 0.073005 78.752619 2.513 0.0140 *  ## poly(years,2)2:plant_richness-0.118095 0.073983 78.206538 -1.596 0.1145  ## ---  ## Signif. codes: 0 ‘***’ 0.001 ‘**’ 0.01 ‘*’ 0.05 ‘.’ 0.1 ‘ ’ 1  ##  ## Correlation of Fixed Effects:  ## (Intr) pl(,2)1 pl(,2)2 plnt_r p(,2)1:  ## ply(yrs,2)1 -0.358  ## ply(yrs,2)2 -0.206 -0.350  ## plnt_rchnss -0.728 0.485 0.099  ## ply(y,2)1:_ 0.293 -0.941 0.299 -0.365  ## ply(y,2)2:_ 0.028 0.413 -0.930 0.162 -0.316  ## Analysis of Deviance Table (Type II Wald chisquare tests)  ##  ## Response: intdiv  ## Chisq Df Pr(>Chisq)  ## poly(years, 2) 43.2650 2 4.028e-10 ***  ## plant_richness 14.4034 1 0.0001475 ***  ## poly(years, 2):plant_richness 7.0309 2 0.0297343 *  ## ---  ## Signif. codes: 0 ‘***’ 0.001 ‘**’ 0.01 ‘*’ 0.05 ‘.’ 0.1 ‘ ’ 1 |
| Network complexity (Connectance) |
| ## Linear mixed model fit by REML. t-tests use Satterthwaite's method  ## ['lmerModLmerTest']  ## Formula: connectance ~ poly(years, 2) + (1 \| replicate) + (1 \| method) + (1 ## \| plot)  ## Data: summaryt_int  ##  ## REML criterion at convergence: -270  ##  ## Scaled residuals:  ## Min 1Q Median 3Q Max  ## -1.2558 -0.8678 0.0194 0.3994 3.9083  ##  ## Random effects:  ## Groups Name Variance Std.Dev.  ## plot (Intercept) 4.569e-05 0.00676  ## replicate (Intercept) 1.303e-03 0.03610  ## method (Intercept) 0.000e+00 0.00000  ## Residual 2.852e-03 0.05341  ## Number of obs: 96, groups: plot, 4; replicate, 3; method, 2  ##  ## Fixed effects:  ## Estimate Std. Error df t value Pr(>\|t\|)  ## (Intercept) 0.06606 0.02181 2.08853 3.029 0.089 .  ## poly(years, 2)1 0.07341 0.05341 88.00000 1.375 0.173  ## poly(years, 2)2 0.05123 0.05341 88.00000 0.959 0.340  ## ---  ## Signif. codes: 0 ‘***’ 0.001 ‘**’ 0.01 ‘*’ 0.05 ‘.’ 0.1 ‘ ’ 1  ##  ## Correlation of Fixed Effects:  ## (Intr) p(,2)1  ## ply(yrs,2)1 0.000  ## ply(yrs,2)2 0.000 0.000  ## optimizer (nloptwrap) convergence code: 0 (OK)  ## boundary (singular) fit: see help('isSingular')  ## Analysis of Deviance Table (Type II Wald chisquare tests)  ##  ## Response: connectance  ## Chisq Df Pr(>Chisq)  ## poly(years, 2) 2.8098 2 0.2454 |

**Figure S1.** Impact of glacier retreat on plant diversity (Shannon index). (A) Effects of glacier retreat (*x-*axis) on plant diveristy (Shannon index, *y*-axis), and (B) on the number of plant species flowering (*y*-axis).

**Figure S2**. Impact of glacier retreat on pollinator communities. (A) Effects of glacier retreat (*x-*axis) on pollinator abundance (number of individual, *y*-axis), and (B) on the number of pollinator diversity (Shannon index, *y*-axis).

**Figure S3**. Effects of glacier retreat (*x-*axis) on the number of plant species visited by pollinator (*y*-axis), and (B) on the frequency of plant species visited by pollinators (*y*-axis).
